# Student health and well-being in secondary schools: the role of school support staff alongside teaching staff

**DOI:** 10.1080/02643944.2018.1528624

**Published:** 2018-10-14

**Authors:** H. J. Littlecott, G. F. Moore, S. M. Murphy

**Affiliations:** Centre for the Development and Evaluation of Complex Interventions for Public Health Improvement (DECIPHer), School of Social Sciences, Cardiff University, Cardiff, Wales, UK

**Keywords:** Support staff, school health, well-being, school relationships

## Abstract

A growing evidence base indicates that health and educational attainment are synergistic goals. Students’ relationships with teachers and other students in the school environment are consistently predictive of a broad range of health and well-being outcomes. Despite the potential importance of relationships between students and a broad range of actors within a school, research tends to reduce ‘school staff’ to ‘teachers’. Previous research has highlighted incongruence between the power imbalance within a teacher–student relationship and the dynamics required to address health and well-being-related issues. To date, there has been no investigation into how the nature of the relationships between students and support staff may differ from those with teaching staff. This article aims to conceptualise the role of support versus teaching staff in promoting health and well-being to understand how school system functioning may affect relationships between school staff and students. Semi-structured interviews were conducted to obtain the perceptions of staff, students and parents within four exploratory case study schools of differing socio-economic status, geographical location and size. In line with the Theory of Health Promoting Schools and Human Functioning, findings demonstrated that the prominence of well-being relies on provision of staffing structures which include a team of support staff to work alongside teaching staff to provide the time and space to deal with issues immediately and build trust and rapport in a one-to-one setting. Further mixed-methods research is required to investigate how staffing structures can facilitate the development of mutually trusting relationships between staff and students.

## Introduction

Adolescent health behaviours such as physical activity, diet and substance use (Boreham et al., 2004) as well as outcomes, such as mental health and subjective well-being (Park, 2004), track into adulthood and are patterned by social and economic characteristics throughout the life course (Moore & Littlecott, 2015a; Moore, Littlecott, Turley, Waters, & Murphy, 2015b). Thus, adolescence is a key life-course period during which to intervene to establish healthier trajectories. Schools provide a setting with high potential reach to facilitate delivery of universal adolescent health interventions which aim both to improve population health and to narrow inequalities (Bonell et al., 2013, 2014a; Langford et al., 2014; Moore et al., 2015b).

Recent systematic reviews have also demonstrated the importance of a positive school environment for students’ well-being. For example, Bonell et al. (2013) found lower rates of substance use where students were more engaged in school. Value added in terms of the extent to which educational outcomes and attendance were better or worse than anticipated given a school's intake was viewed as a proxy for school culture, with authoritative schools more likely to combine a high level of control with a high level of support for students. A review by Fletcher, Bonell and Hargreaves (2008) investigating school effects on drug use found a causal association between modifying the school environment to increase participation, improved relationships and ethos and reduced drug use, particularly for boys. Many of the included studies focused on the effects of the provision of teaching and pastoral support, policies and the school campus.

Students’ relationships with their teachers and with other students in the school environment are consistently predictive of a broad range of health and well-being outcomes (Bonell et al., 2013; Moore et al., 2017; Suldo et al., 2009). Interventions such as INCLUSIVE (Bonell et al., 2014b), a school environment intervention to tackle bullying, attempt to work with the pre-existing functioning of school systems and better orient these school environments towards supporting health. However, a tendency for pedagogic and management practices supportive of student well-being not to become fully integrated into school systems has been linked to pressures from regulatory bodies to attain high levels of academic achievement, coupled with a perception that health and well-being is a competing, rather than synergistic, priority for schools (Elgar et al., 2015; Hanson & Chen, 2007; Viner et al., 2012). Teachers tend to have high workloads leading to the prioritisation of work perceived as directly related to core school business and deprioritisation of work seen as more peripheral to this (Bonell et al., 2014a; Keshavarz, Nutbeam, Rowling, & Khavarpour, 2010).

A growing evidence base, however, indicates that health and educational attainment are synergistic rather than competing goals (Littlecott et al., 2018). Furthermore, education policy has increasingly diverged between the UK nations since devolution; in Wales, a number of recent policy developments have put support for health and well-being at the forefront of what schools are expected to do. The Well-being of Future Generations Act (Welsh Government, 2015) mandated the need for all public bodies to consider health impacts in everything they do. The Donaldson Review (Donaldson, 2015) has triggered ongoing processes of curriculum reform, including health and well-being as one of the six key areas of learning and experience and support for student well-being as one of the key pillars of education against which schools performance will be monitored going forward.

Better supporting health and well-being to achieve the aims of these reforms may require that individual teachers, and schools as a whole, adopt a range of unfamiliar principles into their teaching and management practices. A qualitative study of teacher–student relationships found that the development of close relationships, with more frequent discussion of topics away from the academic subject of study were perceived by teachers to be supportive of students’ mental health (Maelan, Tjomsland, Baklien, Samdal, & Thurston, 2018). However, this study only collected data from teachers. Indeed, to date, much literature on the role of school staff in supporting student well-being has tended to reduce ‘school staff’ to ‘teachers’ and ignore the growing array of support roles within schools and their potential roles in facilitating student well-being (Van Petegem, Aelterman, Van Keer, & Rosseel, 2008). In contrast, several research articles have highlighted incongruence between the power imbalance within a teacher–student relationship and the dynamics required to address health and well-being-related issues, with staff other than classroom teachers perhaps playing important roles in connecting students to their school and supporting well-being (Bishop, Whitear, & Brown, 2001; Pound, Langford, & Campbell, 2016).

Within the UK government, policies such as ‘Every Child Matters’ (UK Government, 2003) promote the creation of new support job roles for and the involvement of teachers in pastoral care within schools (Andrews, 2006). Indeed, in recent years, there has been a proliferation of support roles related to well-being in schools (Edmond & Price, 2009). Support staff include those members of staff who are not teachers, for example, teaching assistants, information and communication technology (ICT)/lab technicians, nurse/medical staff, pastoral support staff, special needs support staff and foreign language assistants. The average secondary school in Wales in 2017 has approximately 125 teaching staff and 35 support staff (including 2–3 pastoral support staff; Statswales, 2017).

To date, there has been no investigation into how the division of well-being roles between teaching and support staff differs between schools, and how these differing models function differently in terms of their outcomes for students. This article aims to conceptualise the role of support staff (defined as any members of staff who are not teachers, for example, teaching assistants, ICT/lab technicians, nurse/medical staff, pastoral support staff, special needs support staff and foreign language assistants) in promoting health and well-being to understand how school system functioning may affect relationships between school staff and students. The research questions to be addressed are as follows:

- In what way do support staff play a role in building relationships with students and supporting health and well-being?
- How do the roles of support staff and teaching staff in building relationships with students differ in terms of their form and interact in their perceived effects on health and well-being?
- How are differing models for allocation of well-being roles perceived by staff, students and students?

## Methods

Data collection was undertaken between October 2014 and April 2015.

### Case study schools

Four exploratory case studies were undertaken. Purposive sampling using replication logic and aiming for maximum variation was used to select four schools, each within different localities in South Wales. These schools were selected to represent differing geographical locations, sizes and socio-economic status (SES; as measured by the Welsh Index of Multiple Deprivation (WIMD)) and stage reached of the Health Promoting Schools scheme (Patton, Bond, Butler, & Glover, 2003; Rothwell et al., 2010; Yin, 2003). Pseudonyms were used to maintain anonymity. Case study characteristics are summarised in Table 1.

**Table 1.** Characteristics of case study schools.

School	No. of students	WIMD score (low score = highest deprivation)	Geographic location	Stage of Health Promoting Schools scheme
Greenfield	<900	Highest 10% (affluent)	Rural	National Quality Award (highest accolade)
Woodlands	>1200	Around median	Welsh Valleys	Stage 1
Highbridge	<700	Lowest 10% (deprived)	Urban	National Quality Award
Oakwood	>1000	Highest 10% (affluent)	Urban	Stage 3

### Semi-structured interviews

Tables 2–4 provide a summary of participant characteristics.

**Table 2.** Characteristics of staff interviewees.

		Greenfield School	Woodlands School	Highbridge School	Oakwood School
Well-being lead	*Role*	PE teacher	Assistant head teacher	Deputy head teacher	Deputy head teacher
	*Age group*	26–35	46–55	46–55	46–55
	*Gender*	Female	Female	Female	Female
Interviewee 2	*Role*	Assistant head for PSE	Food technology teacher	Well-being manager	School nurse
	*Age group*	36–45	26–35	36–45	46–55
	*Gender*	Male	Female	Female	Female
Interviewee 3	*Role*	Healthy Schools coordinator	PE teacher	Behaviour support officer	Head of PSE
	*Age group*	26–35	26–35	36–45	36–45
	*Gender*	Female	Female	Female	Female
Interviewee 4	*Role*	Food technology teacher	Head of science and student voice	Teaching assistant	Senior learning support officer
	*Age group*	36–45	26–35	36–45	46–55
	*Gender*	Female	Female	Female	Female
Interviewee 5	*Role*	Student support manager			
	*Age group*	46–55			
	*Gender*	Female			

PE: physical education; PSE: personal and social education.

**Table 3.** Characteristics of student interviewees.

	Greenfield School	Woodlands School	Highbridge School	Oakwood School
Number of students (*N*)	6	8	8	8
Year groups represented	7, 8, and 9	7, 8, 9, 10 and 11	8 and 9	7, 11 and 12
Male (*N*)	2	6	4	6
Female (*N*)	4	2	4	2
Ethnicity	All White British	7 White British, 1 White Polish	All White British	All White British

**Table 4.** Characteristics of parent interviewees.

	Greenfield School	Woodlands School	Highbridge School	Oakwood School
Number of parents (*N*)	1	4	3	3
Member of school staff (*N*)	1	4	3	0
Male (*N*)	0	0	0	0
Female (*N*)	1	4	3	3
Telephone (*N*)	0	0	0	3

#### Staff interviews

Face-to-face, semi-structured staff interviews were undertaken with 3–5 members of staff or healthy school coordinators per school, including the well-being lead. Staff were purposively selected and recruited via a snowball sampling technique and approached by the well-being lead. Written informed consent was obtained prior to commencing the interview.

#### Student interviews

Semi-structured paired interviews with students aimed to collect the views of students and compare and contrast these with staff perceptions. For each of the four case study schools, between six and eight students participated in three or four paired interviews. Key informants were purposively sampled. Teachers were asked to identify students from the upper school (Years 10 and 11) and lower school (Years 7, 8 and 9) within each case study school. It was requested that teachers identify students who were considered generally healthy and perceived to be involved in activities and decision-making as well as students who were considered relatively unhealthy and/or relatively disengaged from school life. Parents were informed, and opt-out consent was undertaken for these interviews.

#### Parent interviews

Semi-structured interviews with parents aimed to compare and contrast parental perceptions with those of staff and students. Between one and four parents participated in interviews within each case study school. Parents were pragmatically sampled through opportunities presented by the well-being lead in the school. In Greenfield, Woodlands and Highbridge Schools, staff were unable to recruit parents for interviews. Instead, parents who were also members of staff at the school were recruited. In Oakwood School, the school nurse was able to recruit a range of parents who she had liaised with, who were not members of staff at the school. Written informed consent was obtained prior to commencing the interview.

#### Analysis

Coding was conducted using NVivo software. Interviews were analysed using thematic analysis (Braun & Clarke, 2006) with aspects of a grounded theory approach incorporated (Corbin & Strauss, 2014). Inductive open coding was used to develop an initial coding system before comparing and structuring the codes. This involved repeated reading of the transcripts in an active manner. In line with grounded theory, a second scan of the interview transcripts was then undertaken, while actively suppressing any presuppositions about the data, to identify any other possible themes. All codes were then organised into overarching themes and sub-themes. Themes were then reviewed in terms of whether the data extracts fit into each coherent theme and whether the themes and sub-themes accurately represented the overall data set. Alterations were made accordingly, before naming and defining the themes. This was an iterative process, whereby pertinent codes were elaborated upon within future interviews.

## Results

### Integration of well-being into teaching staff and support staff roles: variations between case study schools

The structure of teams for supporting student well-being, and in particular, the extent to which well-being roles were performed by teaching staff and support staff, varied substantially between case study schools. Within Woodlands School, the Assistant Head was described as acting as a lone figurehead for the strategic management of health and well-being-related activity, with individual well-being roles largely allocated to teaching staff. In Oakwood School, the well-being lead role was assigned to a deputy head who worked closely with the school nurse and personal and social education (PSE) teacher to coordinate health and well-being activity. Greenfield (the most affluent school) had a well-being desk and dedicated well-being team, comprised of both teaching and support staff, with the role of well-being lead assigned to a physical education (PE) teacher. Highbridge, the least affluent school, described a multi-agency healthy living team, with several support roles dedicated to health and well-being, the role of well-being lead assigned to a deputy head and an expansive network of external agencies supporting health and well-being.

### Staff–student relationships and well-being: interacting roles of teaching staff and support staff

Students highlighted the importance of teachers in dealing with issues around health and well-being. Students in Woodlands School mainly reported that teaching staff dealt with any issues that they had, though with somewhat mixed perceptions of effectiveness. For example, a student reported that teaching staff liaised effectively with her mother to resolve a bullying issue:

*(…) when I went through a bullying issue with the school before **my Mam rang up the school and they communicated really well** like my Mam explained the situation and what was going on, and **the school sorted it straight away***. *(Woodlands School, Student interview 4, Year 10 boy and Year 11 girl)*

The extent to which students had built rapport with teachers was perceived to be important in students’ decision to approach them with health and well-being issues:

S1: (…) so the teachers caring about the students makes them want to learn more. Because if you’ve got a teacher who dislikes you or acts like they dislike you, you’re not going to want to be friends with them or do what they say. S2: Yeah like I have, **we have only like two teachers that I actually like**. (Woodlands School, Student interview 3, Year 9 girls)

(…) The first person I would speak to is Mrs [name of teacher] because we are both very close to her, she is lovely. (Woodlands School, Student interview 4, Year 10 boy and Year 11 girl)

Students from Greenfield School also suggested that all teaching staff were approachable, and students from Highbridge School suggested that the option was there for whoever students felt most comfortable approaching, either support staff or teaching staff:

Yeah **if a student has a problem all the teachers will make sure that they’ll be able to help** it, even if they’re not in the wellbeing office, teachers in general. (Greenfield School, Student interview 2, Year 9 girls)

Yeah I think and **you can go to whoever you feel comfortable with** so you if you’re more comfortable with one teacher you can go to them instead of someone else. (Highbridge School, Student interview 2, Year 9 girls)

Meanwhile, a student from Oakwood School reported that confiding in teaching staff in the head of year role allowed them to deal with issues before students came to any serious harm:

Say now somebody’s really depressed and they don’t talk about their feelings, they can have a mental breakdown in class and the teachers don’t know what it’s about, but **if they go to the counsellor or [head of year] or [PSE teacher] they sort it out before anything happens**, like self-harming, like. (Oakwood School, Student interview 1, Year 7 and Year 11 boy)

Staff discussed how the presence of teams of support staff may help to increase the time and expertise available to deal with well-being issues that arise for the students. For example, Highbridge School staff emphasised that time pressures on teaching staff made it difficult for them to deal with well-being issues, without support from support staff:

*(…) we have a lot of schools come in to us here to try and remodel what we have in other schools and it’s sort of cottoned on that **you have to have that solid team for it to work in every school** really (…). Obviously **if you haven’t got that type of team then it’s really hard for that teaching member of staff, time-wise, to be able to address all those type of issues**. (Highbridge School*, *well-being lead)*

A positive school ethos was reported to be generated through student awareness of pastoral support structures, often comprising primarily of support staff roles, and spaces to facilitate a quick response to issues, communicating a message to young people that the school cared about their well-being. Schools reported mechanisms to facilitate this through the provision of physical spaces dedicated to well-being in the form of well-being desks, student support centres, pastoral teams and a full-time school nurse with an office. Students demonstrated good awareness of these support systems, where they were in place:

Yeah they do have a Wellbeing Desk and they offer support up there (…) they’re really good to offer support. (Greenfield School, Student interview 2, Year 9 girls)

Many students from Greenfield and Highbridge Schools emphasised that they could approach the well-being team or pastoral staff in their offices and that they were instrumental in dealing with their health and well-being related issues.

it’s really good because **I’ve used the Wellbeing Office and they’re really helpful and supportive** and they won’t let the issue go until it’s all sorted and especially the ones that are big, that are really important. (Greenfield School, Student interview 2, Year 9 girls)

(…) **you could go up to ‘C’ floor and that’s where [Pastoral Support Officer] and [Pastoral Support Officer]’s office is and one of them are always there**. (Highbridge School, Student interview 3, Year 8 girls)

The provision of time and space within support job roles was perceived as important for dealing with well-being issues. In particular, staff from Highbridge School, highlighted that their team structure was used as a model for other schools to aspire to. This involved a Well-being Department and several support staff with well-being-related responsibilities written into their roles for Greenfield and Highbridge Schools:

***The main strengths of the Wellbeing Department is that our staff, so as part of the wellbeing Department***, *(…) I’m part of the team as well and we have our behaviour support leader who’s part of that team and because **everybody comes together, everybody always talks about any issues**. (Greenfield School*, *well-being lead)*

The well-being lead in Greenfield School perceived their well-being department to be particularly effective due to the small size of their school:

*I think the strength of our school is that, for one, **we’re quite a small school really so that most staff know most children** and certainly the wellbeing team, **the new wellbeing team have got a massive overview of every child** really in the school. (Greenfield School*, *well-being lead)*

Despite this, Oakwood School also described creating time and space for teaching staff to promote well-being by assigning each year group to a block where the heads of year (teaching staff) were based at break time with an open-door policy. This meant that students were able to discuss any issues there and then, so they were ‘nipped in the bud’ rather than being allowed to escalate:

*(…) break time the Head of Year works in her office, or she is patrolling the corridor. Right, so the children **if there are any issues, it’s nipped in the bud**. Same, you know, every block has an area, a designated area for the children and I think it is good (…). (Oakwood School*, *well-being lead)*

This was also supported by students from Oakwood School who stated that they felt more comfortable approaching their Head of Year because they did not have to mix with older children to do so.

### The unique roles of support staff in supporting student well-being

While as stated previously, interviewees described important roles for both teaching and support staff (separately and in interaction) in supporting student well-being, the status of support staff as something other than a classroom teacher was perceived as important in facilitating student well-being, particularly in areas of deprivation. The need to get to know young people’s backgrounds in order to identify problems was highlighted by the well-being lead in Highbridge School. The PE teacher from Woodlands School stated that those in the pastoral team were more likely to know personal details about the students and be able to identify assets and challenges within young people’s backgrounds:

I think if you’re in the pastoral team you are more aware of their background, their family life, their health, their situation you know you get to know if they’re eating properly. (Woodlands School, PE teacher)

While teaching staff have 25–30 students in their classrooms at one time, support staff were described as having more capacity to spend one-to-one time with individual children. Meanwhile, a student from Oakwood School reported that they felt comfortable confiding in the specialist PSE teacher. This role may provide a bridging position between teaching and support staff due to the focus on their classes on more holistic aspects of human development, with the PSE teacher technically teaching staff but with a specific remit to focus on well-being as opposed to ‘core’ academic subjects:

Well **our PSE teacher said that if we have any problems with like emotional, then we can come and talk to her** because she is qualified to listen to us and give us advice. (Oakwood School, Student interview 2, Year 7 boys)

Furthermore, these students articulated that the school nurse’s office and the PSE teacher’s classroom were physical spaces where students knew they could go for help. Staff in Oakwood School particularly emphasised the role of the full-time school nurse in comparison to other schools. While most schools have a Local Authority employed school nurse who is not based in the school, Oakwood School had a full-time school nurse. The nurse stated that she sees a minimum of 10 students per day and that being based at the school allows children to get to know her, thus increasing the likelihood that they will confide in her:

It’s very much safeguarding because I am here, **I know the children, they know and trust me and I have this fixed base so they know that every single day I’m going to be here**. (Oakwood School, school nurse)

The school nurse was reported by students to deal with a lot of issues single-handedly in a confidential manner:

S1: she [school nurse] deals with it herself 99% of the time S2: **she won’t like tell anyone else our problems**, she’ll just keep it to herself, unless it’s really bad, then she’ll just tell our Head of Year. (Oakwood School, Student interview 4, Year 7 and Year 11 boys)

This was supported by students within three out of four paired interviews in Oakwood School who perceived the provision of a full-time school nurse to be a privilege that other schools do not benefit from.

The school nurse featured heavily in parent interviews in Oakwood School, who reported her to be helpful in identifying issues and providing someone for their children to confide in and approach for help, particularly when no such individual was available at home:

*(…) **some children can’t talk at home, so they’ve got somebody then at school** that they can go to by having a nurse or anyone in the department they feel that they can go and see somebody else. (Oakwood School, Parent interview 2*, *female*, *not a member of staff)*

The role of relationship-building by support staff in identifying problems and referring students quickly to the relevant outside agencies, if required, was highlighted by the well-being lead in Highbridge School:

*(…) **having individual staff who have the personal attention to detail with each child** so that they can involve the multi-agencies that are required to make sure that that person, you know, is safe and certainly looked after within the family and in the school (Highbridge School*, *well-being lead)*

This provides an example of how the provision of support staff in the school who can work alongside teaching staff to provide the time and space to deal with issues immediately and build trust and rapport with students may increase the prominence of health and well-being in schools.

The importance of these support staff being perceived as specialists in their area was outlined by some students who stated that they felt more supported due to their presence and availability:

Yeah because we know that we have a different person for our problems, so they’re actually specialised in that area. (Oakwood School, Student interview 2, Year 7 boys)

## Discussion

This article explored how the integration of well-being into secondary schools is facilitated by staffing structures which include support staff in the school. These staff work alongside teaching staff to provide the time and space to deal with issues and build trust and rapport in a one-to-one setting with students. Such open relationship-building between students and staff where students view both teaching and support staff as approachable has been shown to be important in students’ satisfaction with school and subsequently connectedness and health outcomes (Samdal, Nutbeam, Wold, & Kannas, 1998). This is congruent with Markham and Aveyard’s Theory of Health Promoting Schools and Human Functioning, which focuses on building open relationships between staff and students as well as creating a separate well-being structure (Markham & Aveyard, 2003). Markham and Aveyard (2003) postulate that schools can support student well-being through manipulating pedagogic and management practices within schools to better connect students with the instructional order (the means of developing knowledge and skills) and the regulatory order (the institutional norms, value and belief system) of their school. From this perspective, individuals are thought to be in a position to choose positive health behaviours and outcomes when their capacity for practical reasoning (i.e. ability to critically perceive reality and view problems and solutions from different perspectives) and affiliation (i.e. possession of shared values and empathetic understanding of others’ orientations to meaning) are supported. Schools can enable students to realise these potentials through organising the instructional and regulatory order of the school in a manner which reduces barriers between staff and students and improves students’ connectedness to one another and to their school (Markham & Aveyard, 2003). Hence, the results of this study demonstrate that support staff can play an important role in the breakdown of barriers between staff and students and increase students’ potential for the realisation of the capacity for affiliation.

Although this study highlighted that both teaching and support staff can be perceived as approachable, the need for students to have a choice, other than teaching staff, over who to approach was emphasised. This may be due to the professional nature of the relationship between teachers and students, whereby teachers are viewed as authority figures, which may make it difficult to disclose sensitive issues (Hargreaves, 2000). This supports the need for the provision of support staff dedicated to health and well-being. Further to this, the main difference in perceptions of teaching staff versus support staff was the contribution of the provision of dedicated time and space for well-being to building trusting relationships with students reported in this study may facilitate the promotion of well-being within the school system. The perception that this time and space allowed for support staff to have an enhanced awareness of students’ backgrounds may facilitate these members of staff in being proactive in tackling health and well-being issues. This may contribute to breaking down boundaries between students and the school (Markham & Aveyard, 2003) and should be further explored.

Moreover, data showed students to perceive the personality of teaching staff and the extent to which they had built rapport and the perceived specialism of support staff to be important factors in their decisions to approach staff with issues. While awareness of the availability of support staff structures was also highlighted as having an impact.

The finding that the least affluent school had the most comprehensive provision of support staff dedicated to well-being is consistent with previous literature, where a higher volume of health improvement activity has been found to be implemented in more deprived schools (Moore, Littlecott, Fletcher, Hewitt, & Murphy, 2016). However, this may lead to an oversight of the more deprived students who are attending more affluent schools who report poorer relationships with school staff (Moore et al., 2017). Thus, work should be undertaken to investigate any role that models for the distribution of well-being support roles within schools may play in mitigating or perpetuating inequalities.

The finding that the school nurse may play a pivotal role in breaking down barriers between school staff, students and parents is aligned with previous research in Sweden, which found the need for school nurses to have knowledge of the organisation, support from other health professionals and knowledge of evidence-based practice (Reuterswärd & Lagerström, 2010). While the merit of school nurses is supported by limited previous research, alongside the need for more quality research on this topic (Wainwright, Thomas, & Jones, 2000), a shortage of nurses has been reported. In the United Kingdom, nurses are usually allocated to several different schools and are, therefore, not based at any particular school on a full-time basis (Hagell, Rigby, & Perrow, 2015). Further mixed-methods research is required to investigate the effect of this role on improving student health and well-being.

### Strengths and limitations

The study benefits from insights from a wide range of perspectives in a sample of schools selected to provide a variety of school backgrounds. However, the nature of qualitative interviews to discuss health and well-being issues perhaps leads to a somewhat pathogenic models of health improvement focused on dealing with ‘problems’, as opposed to prevention through the development of a supportive staffing structure and relationships. The establishment of a positive school ethos at a more holistic level, for example, may act on students’ well-being without their being aware of its effects. Thus, future studies could benefit from a mixed-methods approach to provide more context to the views of stakeholders, although this was beyond the remit of this study. Sampling of staff, students and parents was undertaken through a pragmatic process with reliance on the well-being lead or the school nurse. This may have resulted in recruitment bias, although a broad range of both positive and negative opinions were expressed, suggesting that this was not the case. The school nurse was successful at recruiting three parents of students from Oakwood School. However, due to difficulty with recruitment, parent participants from Greenfield, Woodlands and Highbridge Schools were also members of staff at the school. Thus, these participants are likely to have had more of an insight into school functioning.

### Implications and conclusion

Support staff with responsibility for well-being may play an important and as yet understudied role in orienting school systems towards health improvement. This may help to overcome the lack of time and space and the power dynamics reported by teachers and students to develop trusting relationships between them and deal with well-being-related issues (Keshavarz et al., 2010). Thus, future investigation should employ mixed methods look in more depth at the impact of the quality of both teaching staff and support staff, as well as students’ knowledge of their existence and availability, on the development of mutually trusting relationships with students. Future research should also investigate the potential role of support staff in narrowing inequalities in health and well-being. While quantitative testing of the insights generated by this study, through multi-level social network analyses to understand how different staffing models for health and well-being impact the ethos of the school, and in turn, student well-being could help to establish whether different distributions of well-being roles across schools have implications for school connectedness and well-being. Many early school environment interventions such as the Gatehouse project (Patton et al., 2003) drew largely upon psychological theories such as attachment theory to understand student–teacher relationships on well-being. While useful at the micro-level, such theories pay little attention to how the institutional processes can affect staff–student relationships and offer insufficient traction for understanding system functioning in schools. Thus, it may be useful for future school environment interventions to employ perspectives such as Markham and Aveyard’s theory, which focuses on how institutional processes can affect staff and student relationships. This may also offer sufficient traction to facilitate understanding of system functioning (Hawe, 2015).
